# Metallization, Material Selection, and Bonding of Interconnections for Novel LTCC and HTCC Power Modules

**DOI:** 10.3390/ma15031036

**Published:** 2022-01-28

**Authors:** Aleksander Sešek, Kostja Makarovič

**Affiliations:** 1 Faculty of Electrical Engineering, University of Ljubljana, Tržaška Cesta 25, SI-1000 Ljubljana, Slovenia; 2 KEKO Equipment, Grajski Trg 15, SI-8360 Žužemberk, Slovenia; kostja.makarovic@ijs.si; 3 Jožef Stefan Institute, Jamova Cesta 39, SI-1000 Ljubljana, Slovenia; 4 Centre of Excellence NAMASTE, Jamova Cesta 39, SI-1000 Ljubljana, Slovenia

**Keywords:** power modules, LTCC baseplate, 3D LTCC structures, power bonds

## Abstract

Ceramic baseplates are important elements in the power modules of electric drives. This paper presents low-temperature cofired ceramic (LTCC) and high-temperature cofired ceramic (HTCC) materials for the fabrication of three-dimensional power modules. The silver-based metallization and power module assembly are presented, together with aluminum-based power wire bonding and an industrial procedure to achieve high solderability and bondability. The results of the bond tests using different metallization materials, especially cost-effective ones, are presented, together with the assembly of the power modules. The best results were achieved with Ag metallization and 380 µm Al wire and with Ag–Pd metallization and 25 µm Al wire, both on an LTCC base. The paper concludes with a dual-pulse electrical test of the power modules, which proves the quality of metallization, the type of material selected, and the correctness of the wire bonding and assembly.

## 1. Introduction

Ceramic microsystems are used in applications where chemical, thermal, and mechanical stability are important [1,2,3,4]. These microsystems include chemical microreactors that have chemical, fluidic, heating, and other functions. In multilayer ceramic, buried channels, conductive lines, and vias are added to perform various functions, from fluid mixing to passive and active thermal transfer [1,2,3,4,5,6,7,8,9]. Low-temperature cofired ceramics (LTCCs), and to a lesser degree high-temperature cofired ceramics (HTCCs), have been recognized as suitable materials for the fabrication of such structures [2,3,4,5,6,7,8,9,10]. LTCCs are most commonly glass–ceramic composite materials, while HTCCs are generally alumina with sintering aids [11]. The main difference between them is the sintering temperature, with LTCCs sintered at around 850 °C and HTCCs sintered at around 1600 °C. The drawback of the high sintering temperature of HTCCs is that it cannot be cofired with highly conductive, low-melting-point metals, such as silver, copper, gold, and their alloys. In contrast, LTCCs can be compatible with these metals. 

Ceramic baseplates are used in power modules for electrical motor drives [12]. The most important feature of ceramic is that it withstands high temperatures and has a lower heat resistivity compared to commonly used materials [13]. A second feature is its high electrical isolation, which prevents electrical current from leaking into the ceramic structure and eventually causes a breakdown or short circuit. Most power electronic circuits that use ceramic baseplates are now made of sandwich-like substrates composed of two metal layers brazed on both sides of a ceramic plate. These are known as direct copper bonding (DCB) substrates or active metal braze (AMB) substrates. 

In high-power modules, such as common printed circuit boards (PCBs), the components are soldered on conductive areas called pads. The ceramic layer is, in most cases, composed of alumina-based ceramics (Al_2_O_3_), which offer relatively good thermal and mechanical properties [14]. Instead of alumina, aluminum nitride (AlN) is used in some systems because of its better thermal properties or zirconia-based ceramics because of their better mechanical properties [15]. The electronic elements are connected with metal traces on a ceramic base, but some of the components need to be connected using wire bonding. The metallization for wire bonding is rectangular and connected with traces of the same material. Most producers of LTCCs offer compatible screen-printable metal pastes that are appropriate for wire bonding. Such pastes are made exclusively of pure gold, and the gold wire is bonded from the component to a screen-printed golden pad on the surface of the ceramic [16,17,18]. Contemporary power electronics tend to use aluminum bonds [19], which changes the type of metallization used on the ceramic modules. Additionally, a large amount of heat is dissipated from the bonded power components. Therefore, the thermal management and thermal resistance of materials involved in the thermal dissipation path should be considered.

**Figure 1:**
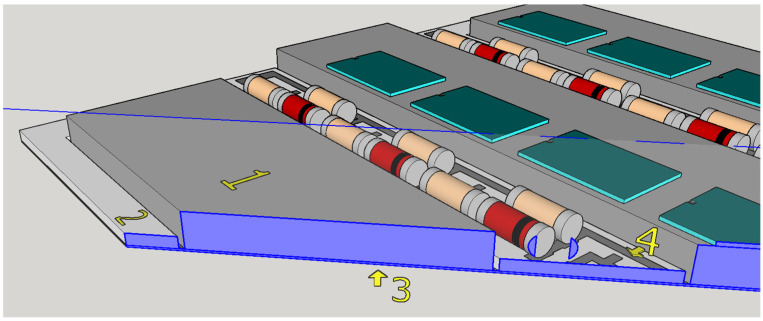
Stacked structure of proposed base plate: (1) NiSn-plated copper busbar, (2) W-shaped second layer, (3) initial bottom base plate, (4) metallization on the top of second layer.

In industrial settings, the price–performance ratio always plays a crucial role. As DCB substrates are low cost and conduct heat easily, they can be used with thicker wire bonding [20]. The most common power module has the planar DCB structure shown in the Figure 1. It has a three-layer copper–alumina–copper structure [21]. LTCC and HTCC materials have not yet been used for such circuits. For this reason, the use of LTCC or HTCC technology, where the conductive lines are screen printed and the structure is made by laminating several layers of ceramics in the unfired state to form a complex 3D circuit, is of great interest. In such circuits, the thickness of the substrate can provide optimized mechanical and thermal management of the circuit. The area with less thickness that fits the busbars where most of the heat is dissipated not only improves the thermal management but also makes the complete circuit planar. This can improve the mechanical reliability of the circuit. The production steps for LTCC and HTCC structures are defined by the user and, as a result, a larger variety and better-optimized structures can be obtained. LTCCs and HTCCs are less complex for fabrication, the whole technological process is cheaper, and it allows cofiring of the ceramics and metal in one process step. Additionally, LTCC and HTCC substrates are compatible with silver and silver–palladium pastes due to the chemical bonds formed. These facts were the motivation for our research work presented in this paper.

The aim of this work is to evaluate the compatibility of high-performance, cost-effective aluminum bonding wires with common screen-printable conductive lines on LTCCs and HTCCs and to evaluate their usability in a high-power electronic module. Several process steps are presented and new approaches in the production steps are described. The adhesion of the metal layers to the ceramics and the bondability of the metallization are evaluated. The electrical test, presented in the final section, confirms that the material selection and their combinations as well as the methods used are appropriate.

## 2. Materials and Methods

### 2.1. Base Plate Fabrication

To produce a 3D ceramic base for the test circuit, SK-47 LTCC tapes and HTCC tapes, both from KEKO-Equipment, Žužemberk, Slovenia, were used. The composition and properties of LTCC are presented elsewhere [22]. The HTCC was based on alumina with sintering aids. The tapes were shaped using a laser drilling and milling machine (LM-8UCC, KEKO-Equipment) and laminated according to the design with an isostatic press (ILS-66, KEKO-Equipment). In the case of LTCC, the metal patterns were printed before firing, while in the case of HTCC, the metal patterns were printed on prefired structures. The LTCC structures were then fired at 850 °C. The same peak temperature was used for firing the printed patterns on the HTCC structures. 

The proposed base plate consists of two stacked layers. The number of layers can be increased later if other shapes are needed. The final stacked structure is presented in Figure 1. The fired dimensions of the bottom base plate are 45 mm × 45 mm, with a thickness of 200 µm (marked with 3). The second layer above is a 500 µm thick, ***W***-shaped plate made from the same material (marked with 2). The electronic elements and connections are placed on the top layer where the printed metal patterns are placed (marked with 4), while the metallization for the busbars’ soldering is prepared 500 µm lower on the exposed first layer. The busbar is a wide copper ribbon up to 3 mm thick (marked with 1). The width of the busbar depends on the power-switching element and is typically 10 mm.

The metal pastes used for the fabrication of the conductive lines are presented in Table 1. The pastes were made by KEKO-Equipment, Žužemberk, Slovenia, and are designed for cofiring with KEKO SK-47 LTCC tape. The pastes were printed on unfired ceramic and then cofired with LTCC, while the same pastes were postfired with HTCC as the melting points of the metals in the pastes are much lower than the sintering temperature of HTCC. Only a few metals are known to be compatible with the soldering and wire-bonding processes. 

**Figure 2:**
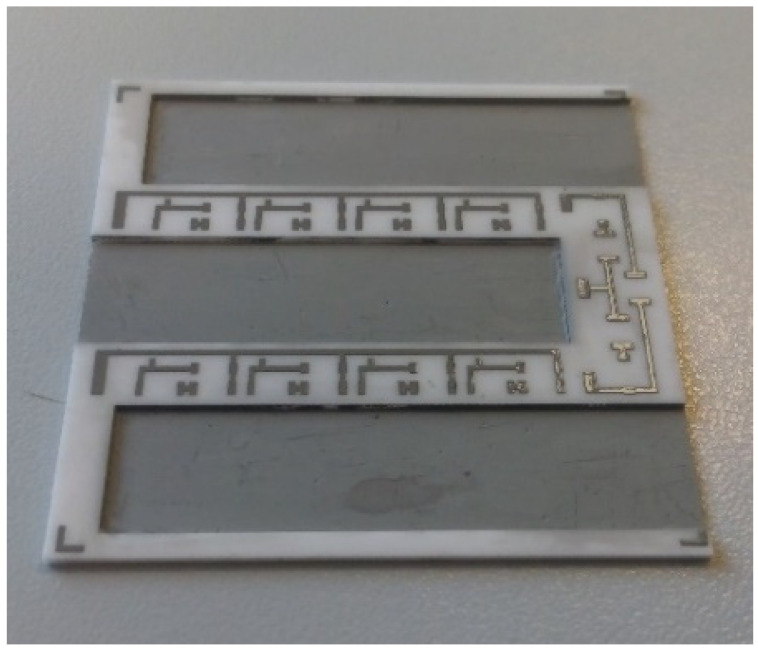
3D HTCC module with Ag–Pd metallization after sintering.

The metals and alloys in Table 1 are suitable for future investigations and tests on ceramic bases. The metals were screen printed using a 325-mesh metal screen, which defines the thickness of the metal layer before drying and firing. The final thickness depends on the solid load of the metal in the paste and can be from a few hundred nm for organometallic pastes up to 100 µm for several consecutive prints. The screen defines the metal lines and shapes for the position of the electronic components and the bonding pads. In Figure 2, a HTCC plate with printed metal lines is shown. In this case, it is a silver–palladium (Ag–Pd) alloy.

This alloy allows a good soldering process, where the solder paste is melted at about 210 °C, while the electric bond is formed between the metal line (Ag–Pd) and the Sn-based contact of the electronic element. This makes it possible to construct the electronic circuit on the ceramic plate. In our case, Alpha^®^ lead-free solder pastes were used with a 210 °C reflow peak temperature [23].

### 2.2. Power Transistor and Bonding

**Figure 3:**
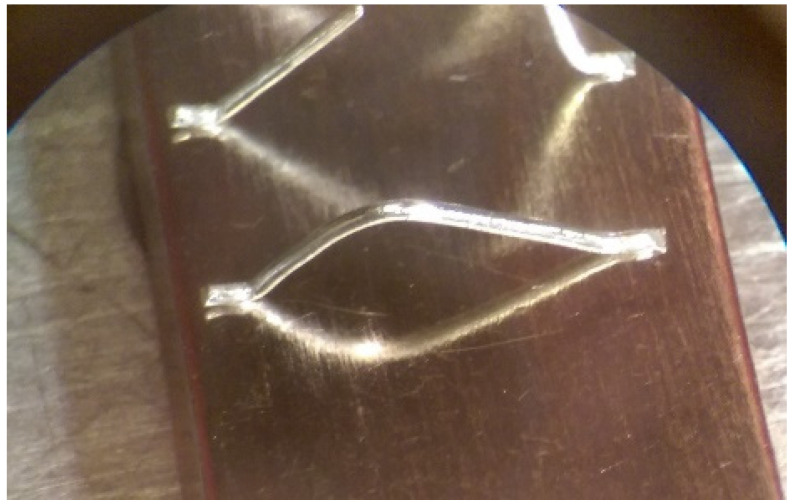
Aluminum bond on a busbar.

As one of the important power electronic components is the bare die of the power transistor [24], another process called bonding is needed. In our case, ultrasound wire bonding was used. During ultrasound wire bonding, the electrical connection between two distant contacts is made using a thin metal wire bonded with ultrasound. It is bonded from the electronic element pad to the contact area on a metalized ceramic plate or a busbar. The bonding test was made using a Delvotec 5310 bonder with 25 µm Al (Hereaus, Al–Si 1%) wire, 25 µm Au (Hereaus, HD2) wire, and a Delvotec 5650 bonder with 380 µm Al wire (Hereaus, Al-H11); the values stated are diameters for all the wires. A power bond example is shown in Figure 3.

The power bond was, in our case, an aluminum wire with a 380 µm diameter [25]. The bond was made on a nickel-plated copper busbar using ultrasound power, pressing force, and sometimes also preheating of the base. All the mentioned factors influence the quality of the electrical and mechanical contacts and define the electrical resistance of the contact. In the case of the busbar, the surface is prepolished and cleaned with 2-propanol, after which the metal is galvanically deposited. In our case, the nickel (Ni) was first galvanically deposited with 25 A of electrical current for 75 min and later tin (Sn) with 30 A of electrical current for 50 min. In this case, the adhesion is high, with the bonds surviving more than 10 N of force used in the pull test [26]. Such a busbar surface is also prepared for soldering of the power component. 

**Figure 4:**
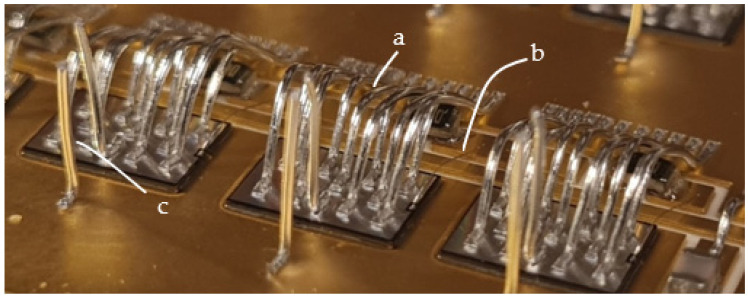
Power MOS transistor on gold-plated copper busbar (a—power bond, b—control bond, c—test bond).

Figure 4 shows power MOS transistors soldered on a gold DCB board busbar bonded with two kinds of wires. The busbar in this case was a 500 µm thick gold-plated copper, which is standard in DCB technology.

Figure 4 shows that besides the power bonds (marked with a), another bond for transistor “control” is present (marked with b) with a diameter of 25 µm. The thin bond is used to flexibly connect the power transistor’s gate pin to the corresponding metal trace on the ceramic base’s connection net. The multitude of thicker 380 µm bonds (a) are needed to conduct a huge amount of current (in this case, up to 100 A per power element) to the phase connection, which is the circuit’s main output. The bonds connect the upper surface of the transistor (source) and the thick copper busbar. Additional vertical bonds on the busbar at the power transistors (marked with c) are used for measurements. For a good electrical contact between the bond and the metal surface, the right material and correct surface treatment must be selected. The DCB plates have a planar design of module base plate. This planar design causes higher arcs of the bonds as they must pass the electronic components and therefore increase the inductances and resistances of the current path. The second issue is the thin copper layer (500 µm) for higher currents and nonoptimal heat dissipation to the cooler beneath (DCB: 500 µm Cu, 500 µm of alumina, and 500 µm Cu), which leads to conduction of electrical current with high losses. In order to increase the power limit, the busbars can be soldered on the copper metallization of the DCB. As the DCB is planar, an additional temporary supportive structure can be added to position the busbars during the reflow process. 

**Figure 5:**
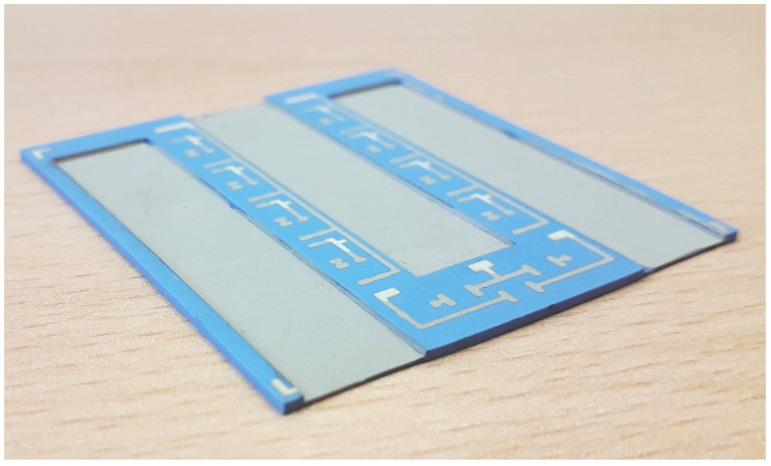
3D LTCC structure with metallization.

In our case, the planar design was replaced with a 3D ceramic structure, shown in Figure 2 (HTCC). Another 3D structure, made from LTCC in this case, is presented in Figure 5.

**Figure 6:**
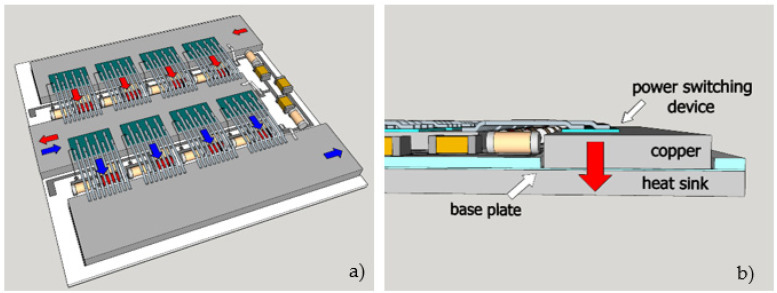
3D model of finalized module (**a**) current flow and (**b**) heat flow.

The current path is solved differently. The soldering paste is deposited on the first base layer and then a NiSn-plated copper busbar is placed and soldered on it. Thick busbars allow higher currents. In our case, the busbar was 2000 µm thick. The thickness of the bottom baseplate can be reduced to 100 µm. The main problem during this step was the use of the correct metal alloy that allows soldering and bonding. With the presented 3D design, the bonding was simplified. The bonds were short and presented lower inductance and resistance as they pass the electronic components without arcs. The heat transfer was, in this case, slightly interrupted with 200 µm of ceramic plate; on the top and bottom are metals (Cu on top and the heat dissipation unit), which have high thermal conductivity. Both the current and the heat flow are presented in Figure 6.

In Figure 6a, the current flow is marked with red (current from a positive supply) and blue (current to a negative supply or ground) arrows. The current path is optimized as the bonding of the power-switching device and NiSn-plated busbar is flattened. Figure 6b presents optimized, vertical heat transfer from the power-switching device to the copper busbar and then through the thin base plate layer with metallization and solder paste to the heat sink. Of course, some of the heat is dissipated through the bonding wires and into the surrounding air. However, the bonding wires are bonded to another busbar, which is at a lower temperature, and the switching device is additionally protected with gel, which lowers the heat dissipation. Therefore, the main current flow is vertical to the bottom heat sink. 

Due to all the mentioned soldering and bonding issues, several steps in the bonding tests and the printing metallization material tests were made.

## 3. Results

The aim of the research was to solve issues associated with introducing new materials and applying new methods in already established industrial procedures.

### 3.1. Soldering Test

**Figure 7:**
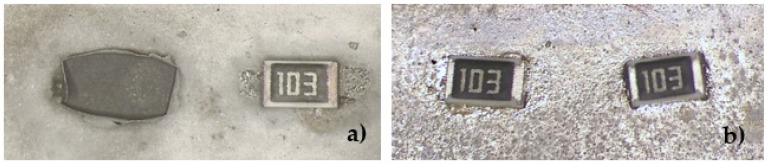
Soldering on metalized ceramic: (**a**) thicker 50 µm metallization with bad adhesion and (**b**) 10 µm metallization with good mechanical and electrical properties.

The soldering test was made with a classical reflow process. It is known that Ag metallization is not compatible with solder paste, while Ag–Pd and Ag–Pd–Pt pastes are dedicated for soldering. Gold paste is not dedicated for soldering, but using it for soldering is possible. The goal was to replace the costly gold paste with silver alloys and find one that would allow bonding with aluminum wire and soldering with Pb-free soldering pastes. The thickness of the metallization printed on the ceramics is the same in all cases. Therefore, approximately the same mass of paste is used. The current price of gold is approximately 60 €/g, while that of silver is around 0.6 €/g [27].The solder thickness was controlled by the screens; it was up to 100 µm. Therefore, the price can be reduced to 1/100 for metallization. The soldering tests were performed after bond testing as bonding is more demanding. The main finding is that the solder joint is stable and electrically highly conductive when the metallization of the ceramic is not thick. In our case, the right thickness was between 10 and 15 µm. For the thicker layers, the electronic elements were soldered to the surface. However, when applying a side force, they were ripped off the surface due to poor adhesion. Figure 7a shows such a case where a 50 µm thick metallization was applied, and Figure 7b shows soldering on 10 µm metallization, which could withstand the mechanical stress.

The different surface colors of the base in Figure 7a,b are due to the thickness of the material and the soldering effect but mainly due to the different angles and illumination settings of the microscope.

Regarding the selection of material, the best for soldering (besides Au) was the Au_50_Pd_35_Pt_15_ paste as it had high reliability and high mechanical stability. The second was Ag_80_Pd_20_, and the worst was pure Ag, which dissolved in solder and was unusable for soldering. The same materials were used for the bond tests, as shown in the next section.

### 3.2. Bond Test

The main bond quality check is made during the bonding procedure when the operator can distinguish good and bad adhesion of the bond wire to the surface from the shape of the bond. However, even if the bond is visually correct, the mechanical bond test is made with a force measurement. This means that a pull test [26] is used at the top point of the bond arc. Here, the measuring head pulls the bond and measures the force when it snaps or when the contact on the metallization breaks off. 

First, the results of wire bonding on the metallization that was printed and postfired on the HTCC tape are presented in Table 2. The table presents ultrasound (US) ball bonding when gold wire was used and US wedge bonding when aluminum wire was used. Each subsequent row shows a microphotograph of the bond with a short description of the pull test result below or a comment. Full comments, an explanation of the remarks, and the issues during bonding are given after the table. However, some general remarks should be explained in advance. The remark “bonding is possible” means that it can be bonded but is not repetitive and robust. “Stable bonding” means it can be bonded with a repetitive process but all the bonds do not pass the pull test. The last remark “bonding is optimal” means that adhesion is strong and pull tests are passed. Unstable bonds are those that can be sometimes made but are not successful in other cases in similar circumstances.

The results from Table 2 show that the 25 µm Al wire can be successfully bonded with Ag metallization on HTCC, while the 380 µm Al wire can be bonded on Ag and 25 µm Al wire to Ag–Pd metallization. The bonding of the 380 µm Al wire to the Ag metallization can result in insulated failures that might be connected to defects on the surface. The Au-printed metallization on HTCCs was not considered because the adhesion is too low. 

In the case of the printed silver pad on the ceramic, there were some issues with the second bond when 25 µm Al wire was used. The adhesion was low when the Ag was not equally thick, especially on the pad’s border areas. The gold wires had bad adhesion with the second bond, where the contact was bad and the repeatability was low.

In the case of the printed Ag_80_Pd_20_, the issues were similar to the Ag metallization. However, in the case of the thick 380 µm Al wires, the adhesion of the metal was even worse as the bonds were loosened and had no contact. 

The last material tested was Au_50_Pd_35_Pt_15_, where only weak 380 µm wire bonds were possible but with low repeatability. 

The results of the bonding tests and their quality on the metalized LTCC plate are given in Table 3, and they show that an adequate quality of bonding was possible. Particularly good was the bonding of the 380 µm wire on the pure Ag metallization. The bonding of the 25 µm Al wire was good on Ag–Pd metallization, while the gold wire was compatible with all except Ag.

In the case of the printed silver pad on the ceramic, there were some issues with the second bond when a 25 µm Al wire was used. As the second bond was a wedge bond, one of the reasons for bad adhesion could be the force used. However, no adjustment gave sufficiently repeatable results. A similar situation occurred when the gold wire was used. The first bond, where the ball bond was used, seemed strong, but the repeatability was low. For the second gold wire bond, the results were even worse.

In the case of the printed Ag_80_Pd_20_ metallization, only slight issues with the second 25 µm Al wire bond appeared, but they were solved with a force adjustment.

The gold-printed pad was good when thin wires, i.e., Al or Au, were used. In the case of the thick Al wire, some of the first bonds were successful, but the majority of the second bonds had poor adhesion, as shown in the figure. The gold peeled off the printed metallization.

The last material tested was Au_50_Pd_35_Pt_15_, where none of the bonding wires was compatible, except the thin Au 25 µm wire, where the bonding had limited quality, as shown by the pull tests.

The bonding samples were checked with microscopes mounted on bonders. On the 25 µm wire bonder, the MOTIC SMZ168 microscope was mounted with a zoom range of 0.5× to 2.0× and a maximum magnification of 20× (with a 10× eyepiece). The second microscope used on the 380 µm bonder was an OLYMPUS SZ30 with a zoom range from 0.9× to 4.0× and a maximum magnification of 80× (with a 20× eyepiece). Figures in the text and tables were taken with different magnification, perspective, and illumination that were the most representative and the best possible for each case.

**Figure 8:**
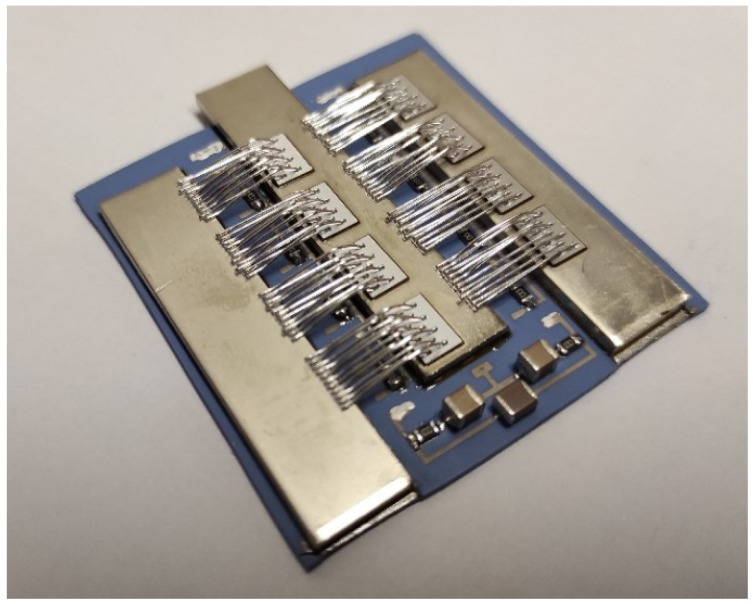
Power module on LTCC 3D base with double metallization.

To meet all the expected boding qualities, bottom Ag–Pd metallization, which enables soldering of the busbars and electronic components, and top Ag metallization, which enables bonding of thin Al wires on a LTCC plate, were combined. This metal combination is shown in Figure 5, where double metallization can be seen. It was used for the final power module assembly shown in Figure 8. 

The final power module with all the steps is presented in Figure 8. For the power switching elements, MOS transistors [24] were used, a gate driver was built in the laboratory, and all the additional electronic components soldered on a ceramic module were chosen to perform the electrical tests under typical conditions presented in the next section. 

### 3.3. Electric Test

**Figure 9:**
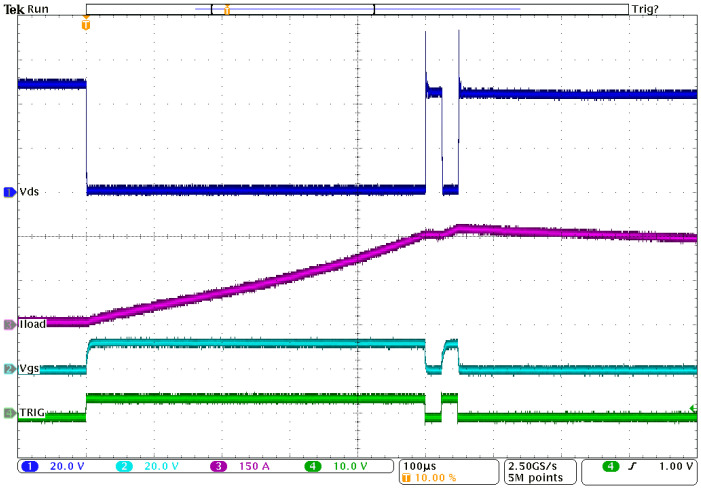
Double-pulse test of power module: input control signal, green trace; voltage on transistor gate, light-blue trace; current through transistor, magenta trace; voltage drop on N-transistor channel, dark blue trace.

To confirm the correctness of the process steps, the electrical performance of the power module was assessed. The power modules were initially tested with a dual-pulse test (DPT) [28,29], where the parasitic influence on the module’s performance was checked. The result is shown in Figure 9.

The electrical test was made using a 48 V supply. For the artificial load, a 60 µH test coil was used as well as an 8000 µF electrolytic capacitor bank. The driver-controlled gate opening at certain time slots is shown on the bottom green trace in Figure 9. The gate voltage is presented on the second light blue trace. From the rise and fall times and their shape, the electronic element’s performance could be seen to influence the switching element’s input capacitance. The transients presented were as expected and corresponded to the time constants set and the driver timings. The dark blue trace on the top presents the voltage on the switching element. From the voltage drop during the “on” state (the longest time slot starting 100 µs after the trigger), the quality of the bonds and the soldering process could be determined. In this case, a small rise in resistance was noticed. It was a similar value to the transistor channel’s resistance (approximately 2 mΩ) depending on the time of observation. The resistance changed due to the heating of system, but the voltage drop did not exceed the expected values in the operating temperature region (up to 90 °C). The exact value of the bond contact could not be measured as the solder paste also had an influence on the total resistance. The magenta trace shows the current value flowing through the transistors and the load. In the first “on” phase, where the current was rising linearly, it reached a value of 300 A. Then, it dropped insignificantly in the 20 µs “off” phase and rose again in the next 20 µs “on” phase to a final value of 330 A. After that phase, the transistor was off, and the energy accumulated in the inductive load slowly decreased. The current in this case was flowing through protective elements (free-wheel diodes) into the supply. From the transient spikes during the second “on/off” phase, the inductive and resistance parasitic could be obtained. In the presented cases, they complied with the expected values calculated from the conductive wires and bonds. The typical resistivity of a single transistor bond joint (12 × 380 µm bonds with a total area of 1.36 mm^2^) was 200 µΩ, while the resistivity of an MOS element is around 2 mΩ. In the case of good bonds, their resistance does not contribute significantly to the overall resistivity, but in the case of poor adhesion of the metal or the bond, the resistance rises and causes a larger power dissipation, leading to future damage to the contact (physical and electrical) and finally to failure. All the issues can be monitored through the phase current and tracking of the dissipated power.

## 4. Summary and Conclusions

LTCCs and HTCCs are used in many industrial areas. An interesting and rapidly developing sector is that of power modules for electric drives. This paper presents one such module, which is initially based on a DCB power module. It has a planar structure with no optimal assembly shape as well with some drawbacks for heat dissipation. The proposed solution presented is a 3D ceramic structure with a LTCC or HTCC metallized ceramic base. This structure can be shaped in the requested way to facilitate assembly and production, minimize inductance of the bonds, and increase thermal conductivity to the heat dissipation unit. The paper concentrates on the silver-based metallization and aluminum wire bonding and presents the results for several metallization materials and different bonding wires. It concludes with an electrical test, confirming that the new assembly steps, material selection, and wire bonding are advantageous, both economically and as an assembly solution. The compatible pastes and materials supported with well-established multilayer technology enables the fabrication of a prototype of an economic, high-power module with improved properties. The best results were achieved with Ag metallization and 380 µm Al wire and with Ag–Pd metallization and 25 µm Al wire on a LTCC base and similar for a HTCC base. The price–performance ratio of such a solution is high, with the presented metalized ceramic base being at least five times cheaper and possessing better thermal and electrical performance. The results show that the methods used for faster industrial production are appropriate, provide high reliability, and economically justify the material used and the bonding.

## Figures and Tables

**Table 1:** Metal fillers and the properties of LTCC-compatible pastes. The pastes can also be used for HTCC but only in postfiring application.

Metal or Alloy	Melting T (°C)	Approx. Sintering T (°C)	Soldering (Lead-Free)	Bonding (Au Wire)	Commercial Paste Name
Ag	961	850	No	NA	KEKO AgL-1
Ag_80_Pd_20_	1020	850	Yes	NA	KEKO AgPdS-1
Au	1063	850	Yes	Yes	KEKO AuB-1
Au_50_Pd_35_Pt_15_	around 1200	850	Yes	NA	KEKO AuPtS-1

**Table 2:** Results of the bond tests on HTCC metallized ceramics.

	Type of Bond Wire	Al 25 µm	Al 380 µm	Au 25 µm
Type of Metallization				
Ag		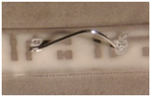	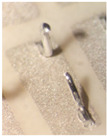	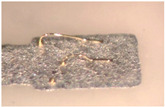
		Pulltest force was around 0.2 N. Some dificulties experienced.	Pull test force was around 7 N.	Unstable bonds.
Ag_80_Pd_20_		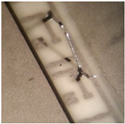	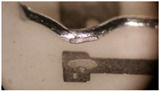	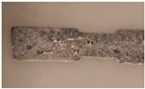
		Pull test force was around 0.2 N. Some dificulties with second bond.	Bonds were unstable. Adhesion of metallization after bonding was limited.	Unstable bonds.
Au_50_Pd_35_Pt_15_		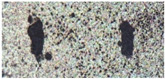	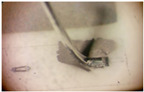	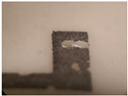
		Adhesion of the metallization to the surface of the HTCC was too low.	Adhesion of the metallization to the surface of the HTCC was too low.	No bonding Possible.

**Table 3:** Results of bond tests on LTCC metallized ceramics.

	Type of Bond Wire	Al 25 µm	Al 380 µm	Au 25 µm
Type of Metallization				
Ag		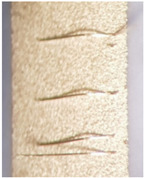	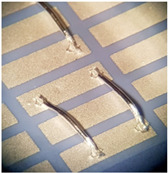	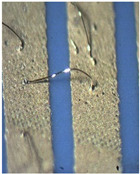
		Pull test force was only 0.1 N. Second bond was unstable.	Pull test force was 6–8 N. Bonding was stable.	Neither first nor second bond was stable.
Ag_80_Pd_20_		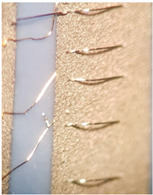	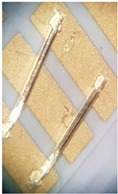	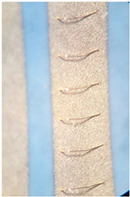
		Pull test force was around 0.2 N.	Pull test force was around 7 N. Bonding was possible.	Pull test force was around 0.5 N. Bonding was stable.
Au		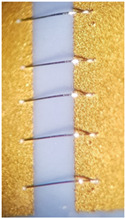	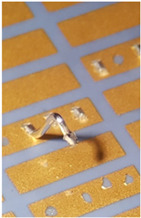	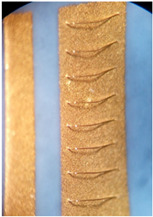
		Pull test force was around 0.6 N. Bonding was possible.	Bonding was not possible. Pull test results not avaliabe.	Pull test force was around 0.6 N. Bonding was optimal.
Au_50_Pd_35_Pt_15_		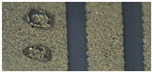	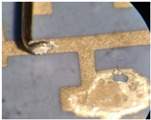	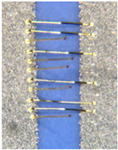
		No bonding possible.	No bonding possible.	Pull test force was around 0.4 N.

## Data Availability

Not applicable.
